# Clinical factors associated with smoking cessation among smokers with Chronic Obstructive Pulmonary Disease by sex: Longitudinal analyses from French smoking cessation services

**DOI:** 10.1016/j.heliyon.2024.e30920

**Published:** 2024-05-09

**Authors:** Ingrid Allagbé, Roche Nicolas, Guillaume Airagnes, Limosin Frédéric, Abdel-Ali Boussadi, Anne-Laurence Le Faou

**Affiliations:** a Centre Ambulatoire d'Addictologie, Hôpital Européen Georges Pompidou, Département Médico-Universitaire de Psychiatrie et Addictologie, AP-HP. Centre - Université Paris Cité, Paris, France; b Respiratory and Intensive Care Medicine, Hôpital Cochin, AP-HP. Centre - Université Paris Cité (EA2511), Paris, France; c UMS 011, Population-based Epidemiological Cohorts, Inserm, Villejuif, France; d Département de Psychiatrie, Hôpital Corentin-Celton, Centre Université Paris Cité, AP-HP, Issy-les-Moulineaux, France; e INSERM, Institut de Psychiatrie et Neurosciences de Paris, Paris, France; f Faculté de Santé, UFR de Médecine, Université Paris Cité, Paris, France; g Département de Santé Publique et Informatique Médicale, Hôpital Européen Georges Pompidou, AP-HP. Centre - Université Paris Cité, Paris, France; h INSERM UMR 1138, Equipe 22, Centre de Recherche des Cordeliers, Paris, France; i Groupement d’Intérêt Scientifique du Réseau Français d’Excellence de Recherche sur le tabac, la nicotine et les produits connexes (REFERtab), Paris, France

**Keywords:** COPD, Tobacco, Smoking cessation, Nicotine replacement therapy, Varenicline, Smoking cessation services

## Abstract

**Background:**

Smoking is responsible for 80 % of cases of Chronic Obstructive Pulmonary Disease (COPD), while the prognosis is improved by smoking cessation (SC). We examined clinical factors associated with SC among smokers with COPD comparing women and men.

**Methods:**

The study comprised a cohort of 1470 smokers who visited a SC service and completed at least 28-day of follow-up visits. The outcome was smoking status at follow-up (abstinence, reduction, no change). Abstinence was defined as continuous abstinence for at least 28 days, validated by the measurement of expired Carbon Monoxide. Reduction was defined as a halving of the baseline tobacco consumption.

**Results:**

The average age of the population was 53 (±11) years and 58.2 % were women. Men were 2 years younger than women and consulted more likely after a hospital contact, whereas women consulted on their own initiative. Women more often had a depression history, whereas men had medical comorbidities and co-addictions. There was no significant difference by sex regarding the abstinence rate (41.0 % in women vs 40.7 in men, p > 0.9). The factors significantly associated with higher abstinence rates in both sexes were: at least one previous quit attempt and number of follow-up visits ≥4. The factors negatively associated with quitting in women were diabetes, intake of mood stabilizers and consuming more than 10 cigarettes per day while having a chronic bronchitis, taking antidepressants and having consumed cannabis in the last 30 days hampered SC in men.

**Conclusions:**

Concerning factors associated with SC, few differences were found between female and male smokers suffering from COPD. However, due to the different medical and smoking behavior characteristics according to sex, it might be important to take these differences into account in order to provide tailored SC management.

## Highlights

• Differences exist between smokers with COPD seeking help to quit according to sex.
• Female smokers had more often a depression history than male.
• Male suffered more often from medical comorbidities compared to women.
• Women smoked less cigarettes per day than men, but their abstinence rate was similar.
• Diabetes hampered cessation in women while cannabis use affected success in men.

## 1 Introduction

Despite the decline in smoking prevalence in France [1], smoking-related morbidity and mortality remain very high, with 75,000 deaths annually and many diseases associated with, or aggravated by tobacco consumption [2]. For instance, between 2000 and 2015, the proportion of deaths attributable to smoking increased by an average of 5.4 % per year among women, compared to a decrease of 1.1 % among men [2]. In 2015, respiratory diseases were the third leading cause of tobacco-related deaths, counting for 16.2 % of them, behind cancers and cardiovascular diseases [2]. Among deaths due to smoking-related respiratory diseases in France, more than 60 % were related to lower respiratory tract diseases such as Chronic Obstructive Pulmonary Disease (COPD) [2]. COPD is an irreversible disease, usually caused by tobacco smoke that substantially impairs health and quality of life [3].

COPD pulmonary complications have increased especially among women while recent COPD prevalence data are lacking in France. Indeed, in 2003, the prevalence of COPD in France was estimated at about 7.5 % among adults aged 40 and over [4]. This prevalence is expected to increase although COPD remains under-diagnosed in France [5]. In addition, between 1998 and 2007, the standardized rate of hospitalization for COPD exacerbations in France increased markedly in women (+4.5 % for women versus +1.5 % for men) [6]. This epidemiological phenomenon is linked to the aging of the generations of women who began smoking in the late 1960s or in the 1970s.

COPD due to tobacco smoking is becoming a public health issue among women [7]. Thus, in the USA, COPD has become the leading cause of death in women [8]. Thus, the EDEN study conducted on 3411 patients at risk of COPD (25 % being women) reported a higher frequency of COPD in women smokers (73.6 %) compared to men smokers (60.1 %) [9]. Although the prevalence of COPD increases with the duration of exposure to tobacco smoke [10], it has been pointed out that with equal tobacco consumption, women are more likely to be hospitalized for COPD exacerbations [11]. As a result, cigarette smoking may have more harmful effects on lung function among women than among men [12]. In addition to smoking, other risk factors such as biomass fuel exposure due to more domestic responsibilities than men, occupational exposure associated to the evolution of women job tasks which are becoming similar to those of men, low socioeconomic status as well as more frequent co-morbidities like asthma and depression, could explain this excess risk of COPD observed in women compared to men [7]. Eventually, there are mechanistic hypothesis trying to explain why women who smoke are more prone to suffer from COPD compared to men such as bronchial hyperresponsiveness and anti-oestrogenic effect of cigarette smoke aggravating airway obstruction [13]. For patients diagnosed with COPD, quitting smoking is among the leading recommendations provided by international guidelines [14]. Women seem to find it harder than men to maintain abstinence [15]. Thus, a large Canadian study indicated that women with COPD who smoke had higher rates of nicotine addiction than their male counterparts [16]. In addition, unlike men, specific obstacles such as fear of weight gain and low self-efficacy to stop smoking persist in women who make a quit attempt [17]. There is also evidence to suggest that women have a significantly faster nicotine metabolism than men, particularly when using oral contraceptives, or depending on the phase of their menstrual cycle, a particular case which may have concerned a number of women in our study [[17], [18], [19]]. Conversely, smoking cessation (SC) leads to greater benefits in women than in men when suffering from COPD [15].

Smoking Cessation Services (SCS) can contribute to reduce smoking prevalence [20]. In France, smokers can access directly to SCS. SCS offer medical and nursing consultations as well as psychological and social support. Thus, given the evolution of the epidemiological data for COPD, we studied the characteristics of women smokers with COPD attending SCS in comparison with those of men. We took advantage of the French national database of SCS (the “Consultation de Dépendance Tabagique” – CDT-net), which registers a large set of variables: sociodemographic and medical characteristics as well as tobacco behaviors and quit attempt outcomes during follow-up.

Regarding the factors associated with SC success and the data of the literature concerning SC among women smokers with COPD, we hypothesized that the smoking cessation rate would be lower in women who smoke compared with male smokers among smokers with COPD visiting SCS. Thus, the objective of this study was to describe the factors associated with SC outcomes by sex^1^ among smokers with COPD followed by the CDT-net cohort.

## 2 Materials and methods

### 2.1 Study design

We performed a longitudinal study with data recorded in the CDT-net computerized database between January 1, 2014, and May 31, 2018.

CDT-net, which was set up in 2001, contains information about the smokers seeking help to quit in SCS. It provides an online anonymized identifier to each new smoker who visits a SCS, participating in this national database. During their first consultation in a SCS, smokers are asked to fill in a standardized national paper questionnaire “Consultation de Dépendance Tabagique- CDT” (available on “Consultation de tabacologie – 2018” in santepubliquefrance.fr)). The filled-in information completed by the smoker is registered by the SCS staff in the CDT-net database with the smoker's consent. Consequently, data concerning smokers who have refused to be registered in CDT-net are not available. The follow-up data and the smoking status are systematically recorded in CDT-net at each consultation. Among CDT-net contributors, 76 % are hospital-based services.

CDT-net has been authorized by the *Commission Nationale de l’Informatique et des Libertés*, the French data protection agency (N° 739406). Research on SCS data is in accordance with the guidelines of the Declaration of Helsinki. Smokers visiting SCS are informed on the collection of data in CDT-net as well as its objectives. Data in the CDT-net database are anonymized and are collected from individuals receiving routine treatment.

### 2.2 Measurement

For the study, the data collected include sociodemographic variables (age, sex, educational level, employment status) and clinical characteristics declared by the smokers during the first consultation such as cardiovascular risk factors (arterial hypertension, diabetes, hypercholesterolemia), cardiovascular diseases (myocardial infarction (MI), angina, stroke and peripheral arterial disease (PAD)), tobacco-related cancers, depression history, together with the psychotropic treatments taken: antidepressants, anxiolytics, hypnotics and since 2015, mood stabilizers and neuroleptics. Concerning respiratory diseases, asthma, chronic bronchitis, and COPD are recorded. Chronic bronchitis is defined as a morning cough occurring at least 3 months per year for 2 years. Patients can answer “yes” or “no”. The diagnosis of COPD is self-reported. Variables, relating to the smoking behavior are detailed, such as the level of tobacco consumption at inclusion and the number of previous attempts to quit lasting more than seven days. The Heaviness of Smoking Index (HSI) is used to assess nicotine dependence. It is a brief measure including two items (time to first cigarette of day and number of daily cigarettes) with a score ≥4 indicating a high nicotine dependence [21]. In addition, the smokers’ confidence in their ability to quit is assessed on a visual analog scale running from 0 to 10 which measures self-efficacy to quit [22]. Self-efficacy to quit is considered low for scores of 0–4, moderate for scores of 5 or 6, and high for scores ≥7. Electronic cigarette use is registered during the first consultation among the participants. Concerning co-addictions, the number of glasses of alcohol consumed per day is recorded with an alcohol use disorder defined by two alcohol units per day for women and men [23]. Furthermore, the consumption of cannabis in the past 30 days and the taking of opioid substitution treatment are also registered. Finally, the number of follow-up consultations after the first consultation is counted and divided into three classes (1–3, 4–6, ≥7). Followed-up people come to a SCS visit every week or 10 days according to the availability of the different team members.

For this study, we included all consecutive current smokers^2^ aged 18 years or older at the time of the first consultation in a SC service, who declared COPD and completed at least 28 days of follow-up in a SCS. Participants who were non-smokers at inclusion were excluded from the study. Data were recorded in the CDT-net computerized database between January 1, 2014, and May 31, 2018.

Independently from the follow-up duration, any followed-up smoker who declared having stopped smoking for at least 28 consecutive days during the study period, with an expired Carbon Monoxide (CO) concentration <10 ppm measured at each monitoring consultation (floating abstinence), was considered to be abstinent, according to criteria used in real-life settings [18,19]. Smokers reducing their consumption during follow-up by at least 50 % relative to levels at the first consultation were considered to have reduced their tobacco consumption [24]. Tobacco consumption of smokers not falling into either of these two groups was considered to be unchanged.

### 2.3 Study population

**Fig. 1:**
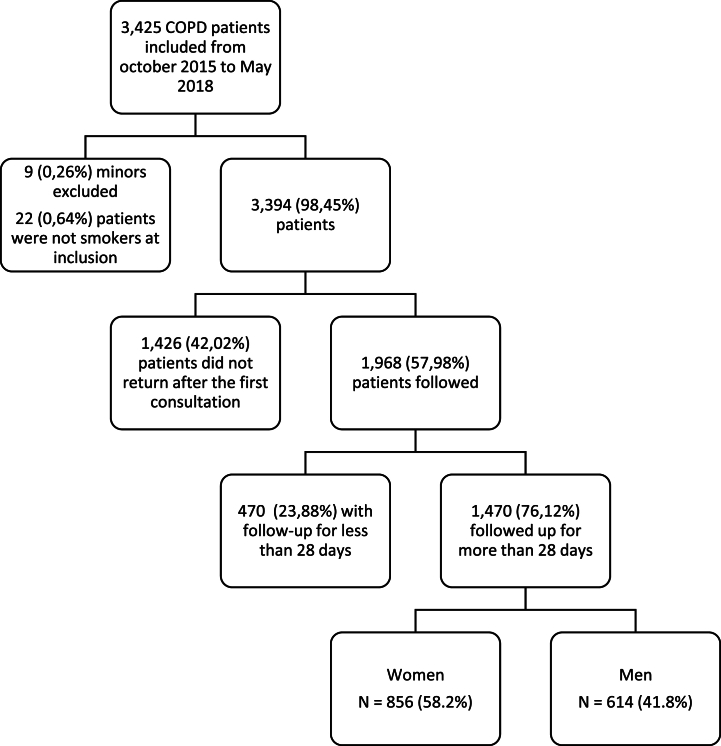
Flowchart of the study population.

Between October 2015, and May 2018, 30,030 smokers were included in the CDT-net database. Overall, 11.4 % (*n* = 3425) self-reported suffering from COPD. A flow-chart of the included population is presented in Fig. 1. About 60 % (*n* = 1968) of the patients returned to SCS after the first visit. Among those returning to SCS and followed for at least 28 days, 76 % (*n* = 1470) performed a median follow-up duration of 59 days for both sexes.

### 2.4 Statistical analysis

All analyses were performed stratified by sex. Student's *t* tests were used for the comparison of continuous variables and Chi2 tests were performed for the comparison of categorical variables. An unconditional bivariate logistic regression analysis was performed, for the variable “smoking status” (abstinence or not), taking into consideration a number of variables of interest (sociodemographic and clinical characteristics, characteristics linked to the smoking behavior and SC treatment prescribed at the first visit). Secondly, a multivariate regression model was performed by stepwise selection. Risk is presented as Odds Ratios (OR) with Confidence Intervals (CI). CI were set at 95 %, with a *p* value < 0.05 considered significant.

Smoking status was described by a binary variable (abstinence: yes/no). All analyzes were performed with R software version 3.5.1.

## 3 Results

The sociodemographic and medical characteristics of the 1470 smokers, as well as their smoking behaviors, co-addictions, and SC treatments prescribed at the first visit are presented in Table 1. The study population consisted of 58.2 % women (*n* = 856), with a mean age of 52 years. About 50 % of the women with COPD were educated to high school level or beyond, and 49 % were employed. The men (41.8 %) had a mean age of 54 years, being slightly older than women and many of them had either no diploma (22 %) or a vocational diploma (35 %). There were some differences between men and women. Notably, women were more likely to use antidepressants and presented more often a depression history compared to men (34 % vs 23 %, p < 0.001). In addition, asthma was more prevalent among women (27 % vs 14 %, p < 0.001). Men were more likely than women to present arterial hypertension, diabetes, high blood cholesterol levels, or cardiovascular diseases. Finally, concerning co-addictions, alcohol use disorder and cannabis use were more frequent among men compared to women.

**Table 1:** Characteristics of patients with COPD included in the CDT-net database and followed from 2014 to 2018.

Variable	Women *N* = 856 (58.2 %)	Men *N* = 614 (41.8 %)	*P*
**Mean age in years (standard deviation)**	52 (11)	54 (12)	<0.001
**Age, *n* (%)**			
18–34 years	77 (9.0)	44 (7.2)	0.007
35–44 years	136 (16)	77 (13)	
45–54 years	273 (32)	167 (27)	
55–64 years	274 (32)	227 (37)	
65–75 years	87 (10)	89 (14)	
≥76 years	9 (1.1)	10 (1.6)	
**Level of education, *n* (%)**			
No diploma	167 (20)	133 (22)	0.029
Vocational school diploma (BEP, CAP)	257 (30)	217 (35)	
High-school level	197 (23)	130 (21)	
Higher education (≥2 years after high school)	235 (27)	134 (22)	
**Employment status, *n* (%)**			
Employed	420 (49)	234 (38)	<0.001
Unemployed^a^	264 (31)	191 (31)	
Retired	172 (20)	189 (31)	
**Medical conditions, *n* (%)**			
Arterial hypertension	151 (18)	145 (24)	0.005
Diabetes	62 (7.2)	64 (10)	0.032
Hypercholesterolemia	158 (18)	147 (24)	0.011
Cardiovascular disease^b^	88 (10)	146 (24)	<0.001
Asthma	233 (27)	83 (14)	<0.001
Chronic bronchitis	74 (8.6)	67 (11)	0.15
Smoking-related cancers^c^	69 (8.1)	56 (9.1)	0.5
Depression history	294 (34)	139 (23)	<0.001
**Pharmacological treatments, *n* (%)**			
Anxiolytics	164 (19)	117 (19)	>0.9
Neuroleptics	76 (8.9)	53 (8.6)	0.9
Hypnotics	110 (13)	64 (10)	0.2
Mood stabilizers	59 (6.9)	33 (5.4)	0.2
Antidepressants	197 (23)	110 (18)	0.018
Opioid substitution treatment	14 (1.6)	10 (1.6)	>0.9
**Reason for consulting, *n* (%)**			
Personal initiative	350 (41)	194 (32)	<0.001
Hospital care	224 (26)	219 (36)	
Encouraged by entourage	47 (5.5)	50 (8.1)	
Referred by health professionals	235 (27)	151 (25)	
**Number of prior attempts to quit, *n* (%)**			
0	244 (29)	200 (33)	0.11
1–2	409 (48)	292 (48)	
≥3	203 (24)	122 (20)	
**Number of cigarettes smoked per day, *n* (%)**			
≤10	202 (24)	100 (16)	<0.001
11–20	348 (40)	204 (34)	
21–40	306 (36)	310 (50)	
**HSI (nicotine dependence), *n* (%)**			
Low: 0-1	98 (11)	72 (12)	0.011
Moderate: 2-3	337 (40)	196 (32)	
High: 4-6	421 (49)	346 (56)	
**Self-efficacy to quit, *n* (%)**			
Low: 0-4	273 (32)	160 (26)	0.041
Moderate: 5-6	324 (38)	242 (39)	
High: 7-10	259 (30)	212 (35)	
**Co-addiction, *n* (%)**			
Cannabis consumption in the last 30 days	81 (9.5)	90 (15)	0.002
≥2 glasses of alcohol per day	162 (19)	206 (34)	<0.001
**Use of electronic cigarettes, *n* (%)**			
E-Cigarette	113 (13)	71 (12)	0.3
**Treatment prescribed at the first consultation, *n* (%)**			
No pharmacological treatment	162 (19)	96 (16)	0.008
Nicotine patch	176 (21)	114 (19)	
Oral form of nicotine	80 (9.3)	63 (10)	
Oral form of nicotine + patch	410 (48)	311 (51)	
Varenicline	18 (2.1)	21 (3.4)	
Varenicline + nicotine substitute(s)	8 (0.9)	8 (1.3)	
Bupropion	2 (0.2)	1 (0.2)	
**Number of follow-up consultations, *n* (%)**			
1–3	482 (56)	351 (57)	0.85
4–6	211 (25)	154 (25)	
≥7	163 (19)	109 (18)	
**Smoking status at last follow-up visit, *n* (%)**			
Abstinence	351 (41.0)	250 (40.7)	>0.9
Reduced consumption	306 (35.7)	220 (35.8)	
No change in status	199 (23.3)	144 (23.5)	

Smokers receiving unemployment benefits, minimal income, disabled allowance and adult handicapped allowance.

a Student, No activity, Invalid/Disabled adult allowance.

b Cardiovascular diseases (myocardial infarction, angina, arteritis of the lower limbs, stroke).

c Lung cancer, otorhinolaryngological cancer, bladder cancer.

The reason for the consultation differed significantly by sex: it was often a personal initiative for women (41 %), whereas the medical management of SC was more likely to be initiated in a hospital environment for men (36 %). One third of men had never tried to quit smoking, whereas this was the case for less than 30 % of the women, with no significant difference. Most of the participants smoked more than 10 cigarettes per day (Cpd), with 50 % of men smoking more than 20 Cpd versus 36 % of women. Men were often more highly addicted to cigarettes, based on HSI score (56 %), than women (49 %). Self-efficacy to quit was lower in women compared to men. Finally, almost 12.0 % of the smokers followed-up in SCS were already using electronic cigarettes at their first visit, with no significant difference between men and women.

The sociodemographic and clinical characteristics of the patients, and their tobacco consumption according to smoking status, are presented in Table 2, comparing women and men. The abstinence rate was 41.0 % for women and 40.7 % for men (p > 0.9). In both men and women, abstinence rates were lowest for unemployed smokers (32 %), those who used neuroleptics or mood stabilizers, who had never tried to quit smoking before (29 % in women vs 33 % in men), who received a treatment of oral form of NRT (21 % in women vs 33 % in men) and those who attended fewer than four follow-up visits (∼32 %) (*p* < 0.05). The proportion of smokers who reduced their tobacco consumption was the same in women and men (36 %). Smokers who were unemployed were more likely to try to reduce their tobacco consumption than to try to quit smoking altogether (38 % of women versus 39 % of men).

**Table 2:** Abstinence rate by profile and sex of smokers with COPD followed during CDT-net consultations for smoking cessation between 2014 and 2018.

Variable	Women *N* = 856 (58.2)				Men *N* = 614 (41.8)				
Status	Abstinence 351 (41.0)	Reduction 306 (35.7)	No change 199 (23.3)	p	Abstinence 250 (40.7)	Reduction 220 (35.8)		No change 144 (23.5)	p
**Mean age in years (standard deviation)**	53 (11)	51 (12)	50 (12)	0.026	55 (12)	54 (11)		53 (12)	0.3
**Age, *n* (%)**				0.26					<0.001
18–34 years	27 (35)	31 (40)	19 (25)		17 (39)	13 (30)		14 (32)	
35–44 years	48 (35)	52 (38)	36 (26)		35 (45)	29 (38)		13 (17)	
45–54 years	105 (38)	104 (38)	64 (23)		53 (32)	73 (44)		41 (25)	
55–64 years	130 (47)	86 (31)	58 (21)		96 (42)	71 (31)		60 (26)	
65–75 years	36 (41)	31 (36)	20 (23)		43 (48)	32 (36)		14 (16)	
≥76 years	5 (56)	2 (22)	2 (22)		6 (60)	2 (20)		2 (20)	
**Level of education, *n* (%)**				0.6					0.10
No diploma	72 (43)	57 (34)	38 (23)		44 (33)	46 (35)		43 (32)	
Vocational qualification	116 (45)	85 (33)	56 (22)		92 (42)	80 (37)		45 (21)	
High-school level	75 (38)	71 (36)	51 (26)		54 (42)	52 (40)		24 (18)	
Higher education (≥2 years after high school)	88 (37)	93 (40)	54 (23)		60 (45)	42 (31)		32 (24)	
**Employment status, *n* (%)**				0.008					0.043
Employed	189 (45)	148 (35)	83 (20)		99 (42)	82 (35)		53 (23)	
Unemployed	86 (33)	101 (38)	77 (29)		62 (32)	74 (39)		55 (29)	
Retired	76 (44)	57 (33)	39 (23)		89 (47)	64 (34)		36 (19)	
**Medical conditions, *n* (%)**									
Arterial hypertension	61 (40)	56 (37)	34 (23)	>0.9	60 (41)	56 (39)		29 (20)	0.5
Diabetes	17 (27)	25 (40)	20 (32)	0.057	25 (39)	24 (38)		15 (23)	>0.9
Hypercholesterolemia	72 (46)	54 (34)	32 (20)	0.4	67 (46)	47 (32)		33 (22)	0.4
Cardiovascular disease^a^	35 (40)	31 (35)	22 (25)	>0.9	60 (41)	49 (34)		37 (25)	0.8
Asthma	101 (43)	75 (32)	57 (24)	0.4	34 (41)	28 (34)		21 (25)	0.9
Chronic bronchitis	30 (41)	31 (42)	13 (18)	0.4	23 (34)	29 (43)		15 (22)	0.4
Smoking-related cancers^b^	29 (42)	23 (33)	17 (25)	>0.9	19 (34)	22 (39)		15 (27)	0.6
Depression history	114 (39)	122 (41)	58 (20)	0.029	51 (37)	50 (36)		38 (27)	0.4
**Pharmacological treatments, *n* (%)**									
Anxiolytics	64 (39)	55 (34)	45 (27)	0.4	37 (32)	47 (40)		33 (28)	0.079
Neuroleptics	20 (26)	27 (36)	29 (38)	0.002	10 (19)	21 (40)		22 (42)	<0.001
Hypnotics	46 (42)	37 (34)	27 (25)	0.9	19 (30)	21 (33)		24 (38)	0.016
Mood stabilizers	15 (25)	25 (42)	19 (32)	0.035	5 (15)	16 (48)		12 (36)	0.008
Antidepressants	69 (35)	75 (38)	53 (27)	0.13	32 (29)	45 (41)		33 (30)	0.020
Opioid substitution treatment	0 (0)	6 (43)	8 (57)	<0.001	3 (30)	5 (50)		2 (20)	0.7
**Reason for consulting, *n* (%)**				0.9					0.8
Personal initiative	142 (41)	127 (36)	81 (23)		86 (44)	66 (34)		42 (22)	
Hospital care	92 (41)	82 (37)	50 (22)		81 (37)	86 (39)		52 (24)	
Encouraged by entourage	24 (51)	13 (28)	10 (21)		21 (42)	17 (34)		12 (24)	
Referred by health professionals	93 (40)	84 (36)	58 (25)		62 (41)	51 (34)		38 (25)	
**Number of prior attempts to quit, *n* (%)**				<0.001					0.091
0	71 (29)	111 (45)	62 (25)		66 (33)	84 (42)		50 (25)	
1–2	186 (45)	137 (33)	86 (21)		128 (44)	98 (34)		66 (23)	
≥3	94 (46)	58 (29)	51 (25)		56 (46)	38 (31)		28 (23)	
**Number of cigarettes smoked per day, *n* (%)**				0.11					0.4
≤10	95 (47)	62 (31)	45 (22)		46 (46)	28 (28)		26 (26)	
11–20	145 (42)	119 (34)	84 (24)		81 (40)	72 (35)		51 (25)	
≥21-40	111 (36)	125 (41)	70 (23)		123 (40)	120 (39)		67 (22)	
**HSI (nicotine dependence), *n* (%)**				0.2					0.2
Low: 0-1	38 (39)	34 (35)	26 (27)		25 (35)	25 (35)		22 (31)	
Moderate: 2-3	151 (45)	106 (31)	80 (24)		91 (46)	66 (34)		39 (20)	
High: 4-6	162 (38)	166 (39)	93 (22)		134 (39)	129 (37)		83 (24)	
**Self-efficacy to quit, *n* (%)**				0.001					<0.001
Low: 0-4	87 (32)	110 (40)	76 (28)		52 (32)	66 (41)		42 (26)	
Moderate: 5-6	136 (42)	120 (37)	68 (21)		87 (36)	96 (40)		59 (24)	
High: 7-10	128 (49)	76 (29)	55 (21)		111 (52)	58 (27)		43 (20)	
**Co-addiction, *n* (%)**									
Cannabis consumption in the last 30 days	28 (35)	32 (40)	21 (26)	0.5	23 (26)	40 (44)		27 (30)	0.007
≥2 glasses of alcohol per day	69 (43)	47 (29)	46 (28)	0.085	82 (40)	70 (34)		54 (26)	0.5
**Use of electronic cigarettes, *n* (%)**				>0.9					0.2
E-Cigarette	47 (42)	40 (35)	26 (23)		25 (35)	23 (32)		23 (32)	
**Treatment prescribed at first visit, *n* (%)**				0.001					0.001
No pharmacological treatment	64 (40)	62 (38)	36 (22)		38 (40)	35 (36)		23 (24)	
Nicotine patch	79 (45)	61 (35)	36 (20)		47 (41)	37 (32)		30 (26)	
Oral form of nicotine	17 (21)	32 (40)	31 (39)		21 (33)	19 (30)		23 (37)	
Oral form of nicotine + patch	175 (43)	144 (35)	91 (22)		127 (41)	121 (39)		63 (20)	
Varenicline	11 (61)	2 (11)	5 (28)		11 (52)	5 (24)		5 (24)	
Varenicline + nicotine substitute(s)	4 (50)	4 (50)	0 (0)		6 (75)	2 (25)		0 (0)	
Bupropion	1 (50)	1 (50)	0 (0)		0 (0)	1 (100)		0 (0)	
**Number of follow-up consultations, *n* (%)**				<0.001					<0.001
1–3	157 (33)	173 (36)	152 (32)		113 (32)	122 (35)		116 (33)	
4–6	100 (47)	76 (36)	35 (17)		76 (49)	59 (38)		19 (12)	
≥7	94 (58)	57 (35)	12 (7.4)		61 (56)	39 (36)		9 (8.3)	

Significant difference for women, significant difference for men.

a Cardiovascular diseases (myocardial infarction, angina, arteritis of the lower limbs, stroke).

b Lung cancer, otorhinolaryngological cancer, bladder cancer.

The factors increasing the chances of abstinence for all patients were: being employed or retired, having made previous attempts to quit smoking, for which a dose-response effect was observed, presenting high levels of self-efficacy to quit, receiving varenicline or varenicline associated to NRT and benefiting from ≥4 follow-up consultations. A significant difference in the effectiveness of SC options was also observed with the association varenicline-NRT, increasing the SC rates among both sexes (Table 3). After adjustment, only three variables were associated positively with SC in women and men: having made at least one previous attempt to quit, high self-efficacy to quit and benefiting from ≥4 follow-up consultations. The factors negatively associated with quitting in women were diabetes (OR = 0.46; IC_95 %_ [0.24–0.87]), intake of mood stabilizers (OR = 0.49; IC_95 %_ [0.24–0.94]) and consuming >10 Cpd. The factors associated negatively in SC in men were: having a chronic bronchitis (OR = 0.55; IC_95 %_ [0.30–0.97]), taking antidepressants (OR = 0.52; IC95 % [0.31–0.86]) and having consumed cannabis in the last 30 days (OR = 0.49; IC95 % [0.28–0.83]) (Table 4).

**Table 3:** Factors associated with abstinence in smokers with COPD attending CDT-net smoking cessation services between 2015 and mid-2018 by sex: univariate analysis.

Variable	Women OR [95 % CI]	*P*	Men OR [95 % CI]	*P*
**Age**				
18–34 years	–		–	
35–44 years	1.01 [0.56–1.83]	0.97	1.32 [0.62–2.85]	0.47
45–54 years	1.16 [0.69–1.98]	0.59	0.74 [0.37–1.49]	0.39
55–64 years	1.67 [1.01–2.85]	0.045	1.16 [0.60–2.29]	0.65
65–75 years	1.31 [0.70–2.48]	0.41	1.48 [0.72–3.14]	0.29
≥76 years	2.31 [0.57–10.04]	0.24	2.38 [0.59–10.51]	0.23
**Level of education**				
No diploma	–		–	
Vocational qualification	1.09 [0.73–1.61]	0.68	1.49 [0.95–2.35]	0.08
High-school level	0.81 [0.53–1.23]	0.33	1.44 [0.87–2.38]	0.16
Higher education (≥2 years after high school	0.79 [0.52–1.18]	0.25	1.64 [1.00–2.70]	0.05
**Employment status**				
Unemployed	–		–	
Employed	1.69 [1.23–2.34]	0.001	1.53 [1.03–2.23]	0.037
Retired	1.64 [1.10–2.44]	0.014	1.85 [1.22–2.82]	0.003
**Medical conditions**				
Arterial hypertension	0.97 [0.68–1.38]	0.87	1.04 [0.71–1.51]	0.85
Diabetes	0.52 [0.29–0.91]	0.026	0.93 [0.54–1.56]	0.78
Hypercholesterolemia	1.25 [0.89–1.78]	0.20	1.30 [0.89–1.89]	0.17
Cardiovascular diseases^a^	0.94 [0.60–1.48]	0.80	1.02 [0.70–1.49]	0.91
Asthma	1.14 [0.84–1.55]	0.39	1.01 [0.63–1.61]	0.96
Chronic bronchitis	0.98 [0.60–1.59]	0.93	0.73 [0.43–1.24]	0.26
Smoking-related cancers^b^	1.05 [0.63–1.72]	0.86	0.73 [0.40–1.28]	0.28
Depression history	0.87 [0.65–1.16]	0.34	0.80 [0.54–1.18]	0.27
**Pharmacological treatments**				
Anxiolytics	0.90 [0.64–1.28]	0.57	0.62 [0.40–0.94]	0.027
Neuroleptics	0.48 [0.29–0.81]	0.007	0.31 [0.15–0.61]	0.001
Hypnotics	1.04 [0.69–1.56]	0.85	0.58 [0.33–1.01]	0.06
Mood stabilizers	0.47 [0.25–0.84]	0.013	0.24 [0.08–0.59]	0.004
Antidepressants	0.72 [0.52–1.00]	0.05	0.54 [0.34–0.83]	0.007
Opioid substitution treatment	NA		0.62 [0.13–2.25]	0.49
**Reason for consulting**				
Personal initiative	–		–	
Hospital care	1.02 [0.73–1.44]	0.91	0.74 [0.50–1.09]	0.12
Encouraged by entourage	1.53 [0.83–2.83]	0.17	0.90 [0.48–1.70]	0.77
Referred by health professionals	0.96 [0.68–1.34]	0.81	0.87 [0.57–1.34]	0.54
**Previous attempts to quit**				
0	–		–	
1–2	2.03 [1.45–2.86]	<0.001	1.58 [1.09–2.31]	0.016
≥3	2.10 [1.42–3.11]	<0.001	1.72 [1.08–1.74]	0.021
**Number of Cpd**				
≤10	–			
11–20	0.80 [0.57–1.14]	0.22	0.77 [0.48–1.25]	0.30
≥21	0.64 [0.44–0.90]	0.016	0.77 [0.49–1.22]	0.26
**HSI (nicotine dependence)**				
High: 4-6	–		–	
Moderate: 2-3	1.30 [0.97–1.74]	0.08	1.37 [0.96–1.96]	0.08
Low: 0-1	1.01 [0.64–1.58]	0.96	0.84 [0.49–1.42]	0.96
**Self-efficacy to quit (0**–**10)**				
Low: 0-4	–		–	
Moderate: 5-6	1.55 [1.11–2.17]	0.011	1.17 [0.77–1.78]	0.48
High 7-10	2.09 [1.47–2.98]	<0.001	2.28 [1.50–3.51]	<0.001
**Co-addictions**				
Cannabis consumption in the last 30 days	0.73 [0.45–1.18]	0.22	0.45 [0.27–0.73]	0.001
≥2 glasses of alcohol per day	1.08 [0.76–1.53]	0.64	0.94 [0.67–1.33]	0.74
**Use of electronic cigarettes**				
Electronic cigarette at first visit	1.03 [0.69–1.53]	0.89	0.77 [0.45–1.28]	0.32
**Treatment at inclusion**				
No pharmacological treatment	–		–	
Nicotine patch	1.25 [0.81–1.93]	0.32	1.07 [0.62–1.87]	0.81
Oral form of nicotine	0.41 [0.22–0.76]	0.005	0.76 [0.39–1.48]	0.43
Oral form of nicotine + patch	1.14 [0.79–1.66]	0.49	1.05 [1.48–2.77]	0.83
Varenicline	2.40 [0.90–6.84]	0.084	1.68 [0.65–4.40]	0.28
Varenicline + NRT	1.53 [0.35–6.68]	0.55	4.57 [1.00–3.24]	0.07
Bupropion	1.53 [0.05–39.18]	0.76	0.00 [NA–]	0.98
**Number of follow-up consultations**				
1–3	–		–	
4–6	2.27 [1.85–2.79]	<0.001	2.05 [1.39–3.03]	<0.001
≥7	4.23 [3.39–5.31]	<0.001	2.68 [1.73–4.17]	<0.001

OR: odds ratio. 95 % CI: 95 % confidence interval.

a Cardiovascular diseases (myocardial infarction, angina, peripheral arterial disease, stroke).

b Lung cancer, otorhinolaryngological cancer, bladder cancer.

**Table 4:** Factors associated with abstinence in smokers with COPD attending CDT-net smoking cessation services between 2015 and mid-2018, by sex: stepwise descending regression analysis.

Variable	Women OR [95 % CI]	*P*	Men OR [95 % CI]	*P*
**Employment status**				
Unemployed	–		–	
Employed			1.63 [1.05–2.55]	0.029
Retired			1.64 [1.04–2.62]	0.036
**Medical conditions**				
Diabetes	0.46 [0.24–0.87]	0.019		
Hypercholesterolemia	1.45 [0.98–2.17]	0.065		
Chronic bronchitis			0.55 [0.30–0.97]	0.043
**Pharmacological treatments**				
Neuroleptics	0.60 [0.32–1.09]	0.10	0.46 [0.20–1.00]	0.057
Mood stabilizers	0.49 [0.24–0.94]	0.038	0.45 [0.14–1.24]	0.15
Antidepressants			0.52 [0.31–0.86]	0.013
**Previous attempts to quit**				
0	–		–	
1–2	1.83 [1.28–2.64]	0.001	1.55 [1.03–2.33]	0.036
≥3	1.92 [1.26–2.93]	0.002	1.69 [1.02–2.82]	0.043
**Number of cigarettes smoked per day**				
≤10	–		–	
11–20	0.67 [0.46–0.99]	0.043		
≥21	0.59 [0.39–0.87]	0.009		
**Self-efficacy to quit**				
Low: 0-4	–		–	
Moderate: 5-6	1.46 [1.01–2.10]	0.045	1.00 [0.63–1.59]	>0.9
High: 7-10	1.93 [1.32–2.83]	<0.001	2.28 [1.44–3.67]	<0.001
**Co-addiction**				
Cannabis consumption in the last 30 days			0.49 [0.28–0.83]	0.010
**Treatment at inclusion**				
No pharmacological treatment	–			
Nicotine patch	1.45 [0.91–2.31]	0.12		
Oral form of nicotine	0.42 [0.21–0.79]	0.009		
Oral form of nicotine + patch	1.35 [0.91–2.03]	0.14		
Varenicline	2.72 [0.93–8.62]	0.073		
Varenicline + nicotine substitute(s)	1.51 [0.32–7.19]	0.6		
Bupropion	1.87 [0.07–48.7]	0.7		
**Number of follow-up consultations**				
1–3				
4–6	1.88 [1.32–2.67]	<0.001	2.32 [1.52–3.54]	<0.001
≥7	2.89 [1.96–4.30]	<0.001	3.81 [2.34–6.28]	<0.001

OR: odds ratio, 95 % CI: 95 % confidence interval.

## 4 Discussion

In this study, the average age of the population was 53 (±11) years and 58.2 % were women. Men were 2 years younger than women and consulted more likely after a hospital contact, whereas women consulted on their own initiative. Women who sought help to quit in SCS were more educated than men and more often in employment. Concerning the medical characteristics, women more often had a depression history, whereas men had medical comorbidities and co-addictions. SC rates were nearly 41 % and did not differ between women and male smokers. At least 4 follow-up consultations with the prescription of nicotine patches, varenicline, or a combination of varenicline and NRT were effective treatments favoring SC, among both, men and women. In the stepwise descending regression analysis, the factors negatively associated with quitting in women were diabetes, intake of mood stabilizers and consuming >10 Cpd while having a chronic bronchitis, taking antidepressants and having consumed cannabis in the last 30 days hampered SC in men.

Concerning socio-demographic data, the larger number of women compared to men, in our population of COPD smokers might be linked to the higher prevalence of smoking among professionally active women with a high level of education in comparison with men (27 % vs 22 %) [25]. Contrary to what was observed in our study, most COPD patients are men. The predominance of female smokers with COPD observed in this study can be explained by the fact that more than half (51.4 %) of the smokers attending SCS are women [26]. In addition, there was a larger-than-expected proportion of the sample who were currently unemployed. In fact, as the SCS are accessible free of charge for the disadvantaged smokers, this percentage is in line with previous research highlighting a higher health service utilization in COPD patients with the lowest socioeconomic status [27].

Smoking behaviors description pointed out that cigarette consumption was higher in our study than in the general population of smokers [28]. Daily cigarette consumption among smokers with COPD and the levels of nicotine dependence for these patients are higher than those of the general population [14].

The 28-day abstinence rate was similar for both sexes, 41 %, despite a lower dependence on tobacco among women than in men. This result was unexpected as the women included in our study had made more previous attempts to quit smoking than the men, the number of previous quit attempts being a key-factor in predicting SC [29]. Women maybe had failed to maintain abstinence and may have chosen to seek help in a SCS to stop smoking for a longer period (>28 days), consistent with the moderate and strong self-efficacy to quit behind these visits. Thus, our data suggest that these women may be multiplying their attempts to stop smoking as a means of consolidating their efforts. This seems to be supported by the observation that women with COPD more frequently consulted on their own initiative than men, who attended SCS more often during or after hospitalization.

As found in our study, abstinence rates for smokers with COPD ranged from 25 % to 52 % between four weeks and five years of follow-up in the literature [[30], [31], [32]]. The SC rates in our study were similar regardless of the initial reasons for consulting, whether on their personal initiative, on the advice of their family and friends, or following referral by a health professional for both sexes or after a hospital contact for women. These results suggest that a key element underlying the success of these smokers with COPD in quitting smoking was access to specialist care in SCS. Following management at a SCS, smokers with COPD can manage to quit, regardless of the profile of associated comorbidities. We found nearly 12 % of current electronic cigarette users among our smokers with COPD. This percentage corresponded to about four times the prevalence of current electronic cigarette use in the general population [33]. These smokers might have encountered difficulties to quit with electronic cigarettes as a smoking cessation aid and sought help in SCS to succeed cessation. Literature in prospective cohorts of COPD smokers has pointed out that electronic cigarette did not help them to quit, maybe because they presented with high nicotine dependence as did the smokers in our study [34].

Identifying associated factors to SC in our population of COPD smokers pointed out that in contrast to men, presenting with diabetes hampered SC among women. In the literature, patients hospitalized with COPD and coexisting diabetes have worse clinical outcomes than patients hospitalized with COPD without diabetes in both sexes [35]. Despite the urgency of SC, several studies point out the difficulty of SC in diabetics despite their knowledge of the negative effects on their health [36,37]. Smoking in these patients may be considered as a way to manage the difficulties associated with diabetes complications or due to the fear of gaining weight while attempting to quit [36,38]. Mood stabilizers prescribed for psychological disorders were also negatively associated with SC in women. Consistent with the literature, these results may be explained by the use of psychotropic medications and the higher prevalence of psychological disorders in women than in men in our study [39]. Psychological disorders, such as bipolar disorder, depression and anxiety, are often considered to be aggravating factors in COPD because they increase the rate of exacerbations and hospitalizations among women [18,40].

In our study, around a third of smokers opted for a reduction in tobacco consumption during the follow-up. Thus, for some smokers, particularly those suffering from smoking-related diseases, a reduction in tobacco consumption may be an option as a preliminary step to smoking cessation [41]. In our results, the female smokers who consumed more than 41 cigarettes per day and the unemployed smokers more frequently reduced their consumption rather than stopping altogether. Heavy smokers with COPD may not have been able to stop smoking completely during the follow-up period. A phase in which tobacco consumption is reduced, with the help of pharmacological treatment, may be an acceptable option for launching an attempt to quit [42].

Our results have some limitations. Unless being consistent with published data on SC among COPD smokers, they cannot be extrapolated to the general population of smokers with COPD. Indeed, we did not have systematically access to pulmonary diagnosis, complementary exams results and therapeutic data of our sample. In addition, we could not determine the duration of lung functional impairment between male and female smokers because CDT-net does not register this kind of specialized medical information. Finally, our study only allowed to obtain short-term abstinence rates in COPD smokers visiting SCS to quit. However, studies have shown that smokers who had been abstinent for four weeks had increased chances of a long-lasting cessation [43]. These limitations do not affect the strengths of the study. One of the main strengths of this study was its power (1470 smokers), with the possibility of a comparison by sex for a large number of sociodemographic, medical, and smoking behavior variables. Our study is original because no real-life studies comparing women and men for such a large number of variables have ever been published, to our knowledge. Consequently, our results focusing on SC among a group of male and female smokers suffering from COPD are on interest for lung specialists to encourage them to systematically offer proactive SC interventions to their patients. In particular, our results highlight the importance of follow-up and of the number of consultations required to help this population of high nicotine dependent smokers with tobacco-related diseases to stop smoking. Finally, self-declared abstinence was validated by systematically measuring CO in exhaled air at all consultations.

Our results suggest that different types of management should be proposed by sex. Intensive management should systematically be offered to men during hospitalization with particularly, screening of cannabis use and a following monitoring after discharge. For women, management should take into account diabetes and psychiatric background to facilitate long-term abstinence. Finally, a reduction of tobacco consumption prior to stopping altogether can be an option as a means of promoting adherence, particularly for smokers with low or moderate self-efficacy to quit. Indeed, only the pro-active management of SC can improve the prognosis of COPD.

## 5 Conclusion

There are differences by sex for smokers with COPD attending SCS, in terms of both medical and smoking behavior characteristics. Based on our findings, it is therefore important to take into consideration these differences in order to provide tailored SC management according to sex for smokers with COPD seeking help to quit smoking in SCS.

## Ethics statement

CDT-net has been authorized by the *Commission Nationale de l’Informatique et des Libertés*, the French data protection agency (N° 739406). Research on SCS data is in accordance with the guidelines of the Declaration of Helsinki. Smokers visiting SCS are informed on the collection of data in CDT-net as well as its objectives. Smokers gave their informed consent to the data collection at the first visit in each SCS. Data in the CDT-net database are anonymized and are collected from individuals receiving routine treatment.

## Funding

This study is supported by research funding from 10.13039/100004319Pfizer. The company was not involved in the decisions relating to data collection or in the analysis or presentation of the results of this work.

## CRediT authorship contribution statement

**Ingrid Allagbé:** Writing – review & editing, Writing – original draft, Validation, Software, Methodology, Formal analysis, Conceptualization. **Roche Nicolas:** Writing – review & editing, Writing – original draft, Visualization, Validation. **Guillaume Airagnes:** Writing – review & editing, Writing – original draft, Validation. **Limosin Frédéric:** Writing – review & editing, Validation. **Abdel-Ali Boussadi:** Validation, Data curation. **Anne-Laurence Le Faou:** Writing – review & editing, Writing – original draft, Visualization, Validation, Resources, Methodology, Funding acquisition, Data curation, Conceptualization.

## Declaration of competing interest

The authors declare the following financial interests/personal relationships which may be considered as potential competing interests:Allagbé Ingrid: Pfizer grant for research and communication on CDTnet data. Roche Nicolas: Reports grants and personal fees from Boehringer Ingelheim, Pfizer and Novartis, personal fees from Teva, GSK, AstraZeneca, Chiesi, Mundipharma, Cipla, Sanofi, Sandoz, 3 M, Zambon, outside the submitted work. Airagnes Guillaume: received fees from Lundbeck, Pierre Fabre and Pfizer, unrelated to the article. Limosin Frédéric: received fees from Lundbeck, unrelated to the article. Le Faou Anne-Laurence: conference fees in a conference organized by Pfizer in 2021, unrelated to the article.

If there are other authors, they declare that they have no known competing financial interests or personal relationships that could have appeared to influence the work reported in this paper.

## Abbreviations

Cpd: Cigarettes per day
COPD: Chronic Obstructive Pulmonary Disease
SC: smoking cessation
SCS: smoking cessation services
CDT: “Consultation de Dépendance Tabagique”
MI: Myocardial Infarction
PAD: Peripheral Arterial Disease
HSI: Heaviness of Smoking Index
NRT: Nicotine Replacement Therapy
OR: Odds Ratios
CI: Confidence Intervals
CO: Carbon Monoxide

## Footnotes

1 For this study, sex was considered a binary term, “female” or “male” and smokers reported their self-representation as male or female.

2 Current cigarette smokers were defined as adults who reported smoking combustible tobacco (cigarillo, rolled cigarette, manufactured cigarette) “every day” or “some days” at the time of the interview [44].
