# Supplementary material for: Clinical factors associated with smoking cessation among smokers with Chronic Obstructive Pulmonary Disease by sex: Longitudinal analyses from French smoking cessation services
Source: Heliyon. 2024 May 9;10(10):e30920. doi: 10.1016/j.heliyon.2024.e30920 (PMC11103529; doi:10.1016/j.heliyon.2024.e30920)
Supplement: Multimedia component 1 [file mmc1.pdf]

Consultation de tabacologie

Nom ______________________________

Prénom ______________________________

Adresse ______________________________

____

Code postal |_|_|_|_|_| Ville ______________________________

Téléphone ______________________________ (où vous êtes facilement joignable)

Téléphone portable ______________________________

Vous voulez essayer d'arrêter de fumer

Pour vous accompagner dans cette démarche, le médecin qui va vous recevoir en consultation a besoin de connaître vos habitudes de fumeur. Merci de bien vouloir prendre le temps de répondre aux questions des pages suivantes : elles vous aideront à faire le point et faciliteront le dialogue avec le médecin.

Vous pouvez obtenir plus d'informations sur l'efficacité des consultations de tabacologie en allant sur le site internet de l'équipe de recherche CDTnet **https://cdtnet.spim.jussieu.fr**

Les informations contenues dans ces questions pourront faire l'objet d'un traitement informatique. Conformément aux articles 26 et 27 de la loi 78-17 du 6 janvier 1978 "Informatique et Liberté" et à son décret d'application n°78-774 du 17 juillet 1978, vous disposez d'un droit d'accès, de modification, de rectification et de suppression de ces données. Pour l'exercer, adressez-vous à votre centre de consultation de tabacologie.

Liberté • Égalité • Fraternité
République Française
Ministère chargé de la Santé
Sécurité sociale L'Assurance Maladie
Société Française de Tabacologie
Respadd Réseau de prévention des addictions
www.inpes.sante.fr inpes Institut national de prévention et d'éducation pour la santé

Date : |_|_| |_|_| |_|_|
jour mois année

1 - Pour le rendez-vous d'aujourd'hui, depuis combien de jours avez-vous pris rendez-vous ? |_|_| jours

2 - Date de naissance : |_|_|_|_|_|_|_| 3 - Sexe : H ☐ - F ☐

4 - Taille : ________ cm 5 - Poids : _________ kg

Pour les femmes :

6 - Êtes-vous actuellement enceinte ? Oui ☐ - Non ☐

7 - Si oui, à quel trimestre de grossesse êtes-vous ? 1 ☐ 2 ☐ 3 ☐

8 - Prenez-vous la pilule ? Oui ☐ - Non ☐

9 - Quel est votre objectif en venant en consultation de tabacologie ?

☐ Je voudrais arrêter de fumer complètement
☐ Je voudrais réduire ma consommation de tabac
☐ Je voudrais simplement obtenir des renseignements sur le sevrage tabagique
☐ Je viens d'arrêter de fumer : j'ai besoin d'aide pour maintenir l'arrêt.

Le saviez-vous ? Les résultats obtenus auprès de fumeurs qui ont consulté au moins 3 fois sont : ceux qui ont arrêté du jour au lendemain ont des taux d'arrêt comparables à ceux qui ont réduit progressivement avant l'arrêt complet pendant leur prise en charge en tabacologie.
Plus d'informations sur le site https://cdtnet.spim.jussieu.fr

10 - Quelle est votre situation professionnelle actuelle ?

☐ *Actif* ☐ *Au chômage, bénéficiaire du RSA* ☐ *En formation/Étudiant*
☐ *Retraité* ☐ *Homme ou femme au foyer* ☐ *Invalidité / Allocation adulte handicapé*

Fumez-vous à l'intérieur de votre habitation ? Oui ☐ - Non ☐

Y a-t-il d'autres fumeurs que vous dans votre foyer ? Oui ☐ - Non ☐

11 - Quel est votre niveau d'études ?

☐ *Sans diplôme* ☐ *Niveau secondaire (lycée)* ☐ *Bac +2*
☐ *C.A.P., B.E.P.* ☐ *Baccalauréat* ☐ *Au-delà de Bac +2*

Qui vous a conseillé de venir à cette consultation ? *(Choisissez une seule réponse)*

☐ *Hospitalisation (avant, pendant, après, médecin hospitalier)*
☐ *Demande de l'entourage*
☐ *Médecin traitant*
☐ *Médecin spécialiste*
☐ *Médecin du travail*
☐ *Pharmacien*
☐ *"Tabac info service"*
☐ *Aucune de ces personnes/institutions, c'est une démarche personnelle*

Maladies et traitements

Si vous avez des doutes concernant les réponses aux questions suivantes, n'hésitez pas à en parler à l'infirmier ou au médecin.

12 - Avez-vous un ou plusieurs des facteurs de risque cardiovasculaire suivants ?

☐ Hypertension artérielle ☐ Diabète ☐ Excès de cholestérol

13 - Êtes-vous ou avez-vous été soigné(e) pour une ou plusieurs des maladies cardiovasculaires suivantes ?
- ☐ Infarctus du myocarde
- ☐ Angine de poitrine (angor)
- ☐ Accident vasculaire cérébral
- ☐ Artériopathie oblitérante des membres inférieurs (artérite)

14 - Êtes-vous ou avez-vous été soigné(e) pour l'une des maladies respiratoires suivantes ?
- ☐ Bronchite chronique *(toux le matin au moins 3 mois par an depuis 2 ans)*
- ☐ Bronchopathie chronique obstructive
- ☐ Asthme

15 - Êtes-vous ou avez-vous été soigné(e) pour un ou plusieurs des cancers suivants ?
- ☐ Poumon ☐ Vessie
- ☐ Gorge (ORL) ☐ Un autre cancer (précisez) ______________________________

16 - Prenez-vous assez régulièrement un ou plusieurs des médicaments suivants ?
- ☐ Tranquillisants ☐ Somnifères ☐ Antidépresseurs
- ☐ Neuroleptiques ☐ Régulateurs de l'humeur
- ☐ Traitements de substitution (subutex / méthadone)

17 - Avez-vous déjà été ou êtes-vous soigné(e) pour dépression nerveuse ayant occasionné un arrêt partiel ou total de l'activité ? Oui ☐ - Non ☐

Avez-vous ou avez-vous eu d'autres problèmes de santé que vous souhaitez nous signaler ?

____

____

____

Bilan tabagique

18 - Avez-vous réduit votre consommation de cigarettes au cours du mois précédant cette consultation d'aide à l'arrêt Oui ☐ - Non ☐

19 - **Vous ne fumez pas actuellement**
Depuis combien de temps avez-vous arrêté ? en jours _______ en mois _______

20 - Combien de cigarettes fumiez-vous par jour avant cet arrêt ? _____

21 - **Vous fumez encore actuellement**
Fumez-vous tous les jours ? Oui ☐ - Non ☐

22 - Combien en moyenne fumez-vous chaque jour de :
Cigarettes manufacturées achetées en paquet ? ___ Cigarettes roulées ? ___ Cigarillos ? ___

23 - Fumez-vous ou consommez-vous les produits suivants *(cochez les cases correspondantes)* :
☐ Cigare ☐ Pipe ☐ Tabac à mâcher ☐ Snus ☐ Narguilé / Chicha ☐ Ploom
Autres produits, précisez _________________

24 - Utilisez-vous une cigarette électronique ? Oui ☐ - Non ☐

Pour les utilisateurs de cigarettes électroniques

25 - Quel est le volume de liquide "vapoté" par semaine ? ___________

26 - Utilisez-vous parfois des cartouches de nicotine ? Oui ☐ - Non ☐

27 - Si oui : Quel est le dosage des cartouches de nicotine que vous utilisez ?

Nous vous invitons à partager votre expérience de "vapotage" sur le site internet https://cdtnet.spim.jussieu.fr afin de contribuer à améliorer les connaissances à ce sujet.

Degré de dépendance au tabac *(Merci d'entourer le chiffre qui correspond à votre réponse)*

28 - Le matin, combien de temps après être réveillé(e) fumez-vous votre première cigarette ?

Dans les 5 minutes	3
6 - 30 minutes	2
31 - 60 minutes	1
Plus de 60 minutes	0

29 - Combien de cigarettes fumez-vous par jour, en moyenne ? *(1 cigarillo = 2 cigarettes)*

10 ou moins	0
11 à 20	1
21 à 30	2
31 ou plus	3

30 - Faites le total de vos réponses : Total : ☐

Histoire avec le tabac

31 - À quel âge avez-vous commencé à fumer quotidiennement ? à |_|_| ans

Pour les personnes qui ont déjà essayé d'arrêter de fumer au moins 7 jours dans le passé

32 - Combien de fois avez-vous déjà arrêté de fumer complètement pendant au moins 7 jours (aucune cigarette) ? ____________________

De quelle manière avez-vous arrêté de fumer pendant cette semaine d'arrêt ?

____

____

____

____

33 - Lorsque vous avez arrêté de fumer complètement pendant au moins 7 jours, avez-vous pris du poids ? Oui ☐ - Non ☐

34 - Si oui, combien de kilos ? ____________________

Combien de temps a duré votre arrêt le plus long ? ____________________

Quelle est la date de votre dernière tentative ? ____________________

Quels troubles avez-vous ressentis à l'arrêt du tabac ?

☐ Aucun
☐ Pulsions à fumer
☐ Irritabilité, colère
☐ Agitation, nervosité
☐ Anxiété
☐ Tendance dépressive
☐ Difficultés de concentration
☐ Troubles du sommeil
☐ Augmentation de l'appétit
☐ Autres

Lesquels ? ____________________

____

Pour quelle(s) raison(s) pensez-vous avoir recommencé à fumer ? *(par ordre d'importance)*

1 - ____________________

2 - ____________________

3 - ____________________

4 - ____________________

Pourquoi fumez-vous ?

Évaluez par une note de 0 à 10 les raisons qui vous poussent à fumer. *Lisez chaque proposition et entourez le chiffre qui correspond à votre réponse : 0 signifie "Jamais" et 10 signifie "Toujours".*

35 - Je fume… :	Jamais										Toujours
- parce que c'est un geste automatique	0	1	2	3	4	5	6	7	8	9	10
- par convivialité	0	1	2	3	4	5	6	7	8	9	10
- pour le plaisir	0	1	2	3	4	5	6	7	8	9	10
- pour combattre le stress	0	1	2	3	4	5	6	7	8	9	10
- pour mieux me concentrer	0	1	2	3	4	5	6	7	8	9	10
- pour me soutenir le moral	0	1	2	3	4	5	6	7	8	9	10
- pour ne pas grossir	0	1	2	3	4	5	6	7	8	9	10

Quel est votre degré de motivation ?

36 - Parmi les phrases ci-dessous, laquelle vous décrit le mieux ?

1 « je ne veux pas arrêter de fumer »

2 « je pense que je devrais arrêter de fumer, mais je ne le souhaite pas vraiment »

3 « je veux arrêter de fumer, mais je n'ai pas réfléchi au moment où je le ferai »

4 « je veux réellement arrêter de fumer, mais je ne sais pas quand je le ferai »

5 « je veux arrêter de fumer et souhaite le faire bientôt »

6 « je veux réellement arrêter de fumer, et j'envisage de le faire au cours du trimestre à venir »

7 « je veux réellement arrêter de fumer, et j'envisage de le faire dans le mois qui vient »

37 - Si vous décidiez aujourd'hui d'arrêter de fumer, quelle confiance auriez-vous dans votre capacité à y parvenir ? *Entourez le chiffre qui correspond le mieux à votre situation* :
(0 signifie : "Aucune confiance" et 10 signifie "Totale confiance").

aucune confiance — *totale confiance*

0 1 2 3 4 5 6 7 8 9 10

38 - **Pourquoi voulez-vous arrêter de fumer ?**

« J'aimerais bien arrêter de fumer parce que je voudrais... » ____________________

39 - **Que craignez-vous en arrêtant de fumer ?**

« En essayant d'arrêter de fumer, j'ai peur de... » ____________________

Vos habitudes de vie

40 - Indiquez à quel point vous craignez de prendre du poids si vous arrêtez de fumer :

pas du tout … *extrêmement*

0 1 2 3 4 5 6 7 8 9 10

41 - Indiquez à quel point vous avez confiance en vous pour ne pas prendre du poids si vous arrêtez de fumer :

je n'ai aucune confiance en moi … *j'ai totalement confiance en moi*

0 1 2 3 4 5 6 7 8 9 10

Votre consommation d'alcool

Ce questionnaire concerne votre consommation d'alcool de l'année écoulée et pas seulement celle des dernières semaines. Merci d'entourer le chiffre qui correspond à votre réponse.

Combien de fois vous arrive-t-il de consommer de l'alcool ?

- Jamais ______ 0
- 1 fois par mois ______ 1
- 2 à 4 fois par mois ______ 2
- 2 à 3 fois par semaine ______ 3
- 4 fois ou plus par semaine ______ 4

Combien de verres standard buvez-vous au cours d'une journée ordinaire où vous buvez de l'alcool ?

- 1 ou 2 ______ 0
- 3 ou 4 ______ 1
- 5 ou 6 ______ 2
- 7 à 9 ______ 3
- 10 ou plus ______ 4

Au cours d'une même occasion, combien de fois vous arrive-t-il de boire 6 verres standard ou plus ?

- jamais ______ 0
- moins de 1 fois par mois ______ 1
- 1 fois par mois ______ 2
- 1 fois par semaine ______ 3
- chaque jour ou presque ______ 4

42 - Faites le total de vos réponses ______

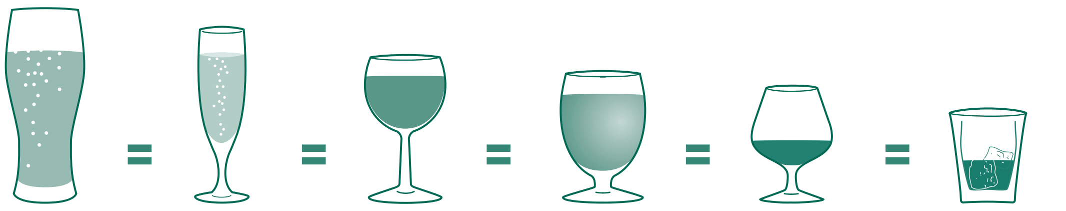

1/2 de bière 5° (25 cl) = Coupe de champagne 12° (10 cl) = Ballon de vin 12° (10 cl) = Verre de pastis 45° (3 cl) = Verre d'apéritif 18° (7 cl) = Verre de whisky 40° (3 cl)

Consommation de cannabis

43 - Avez-vous fumé du cannabis au cours des **12 derniers mois** ? Oui ☐ - Non ☐

44 - Si oui, combien de fois en avez-vous fumé au cours des **30 derniers jours** ?

☐ aucune fois ☐ 1 ou 2 fois ☐ entre 3 et 5 fois ☐ entre 6 et 9 fois

☐ entre 10 et 19 fois ☐ entre 20 et 29 fois ☐ tous les jours

A quel âge avez-vous commencé ? ______

Si vous consommez d'autres produits ou substances, n'hésitez pas à en parler à votre médecin.

Comment vous sentez-vous ?

Actuellement, rencontrez-vous des difficultés particulières dans votre vie professionnelle ?

____

Actuellement, rencontrez-vous des difficultés particulières dans votre vie familiale ?

____

Ce questionnaire a pour but de nous aider à mieux percevoir ce que vous ressentez.
Lisez chaque question et entourez la réponse qui convient le mieux à ce que vous avez ressenti ces derniers jours.
Donnez une réponse rapide : votre réaction immédiate est celle qui correspond le mieux à votre état actuel.

1 Je me sens tendu(e) ou énervé(e) :
- La plupart du temps 3
- Souvent 2
- De temps en temps 1
- Jamais 0

2. Je prends plaisir aux mêmes choses qu'autrefois :
- Oui, tout autant 0
- Pas autant 1
- Un peu seulement 2
- Presque plus 3

3 J'ai une sensation de peur comme si quelque chose d'horrible allait m'arriver :
- Oui, très nettement 3
- Oui, mais ce n'est pas trop grave 2
- Un peu, mais cela ne m'inquiète pas 1
- Pas du tout 0

4. Je ris facilement et vois le bon côté des choses :
- Autant que par le passé 0
- Plus autant qu'avant 1
- Vraiment moins qu'avant 2
- Plus du tout 3

5 Je me fais du souci :
- Très souvent 3
- Assez souvent 2
- Occasionnellement 1
- Très occasionnellement 0

6. Je suis de bonne humeur :
- Jamais 3
- Rarement 2
- Assez souvent 1
- La plupart du temps 0

7 Je peux rester tranquillement assis(e) à ne rien faire et me sentir décontracté(e) :
- Oui, quoi qu'il arrive 0
- Oui, en général 1
- Rarement 2
- Jamais 3

8. J'ai l'impression de fonctionner au ralenti :
- Presque toujours 3
- Très souvent 2
- Parfois 1
- Jamais 0

9 J'éprouve des sensations de peur et j'ai l'estomac noué :
- Jamais 0
- Parfois 1
- Assez souvent 2
- Très souvent 3

10. Je ne m'intéresse plus à mon apparence :
- Plus du tout 3
- Je n'y accorde pas autant d'attention que je devrais 2
- Il se peut que je n'y fasse plus autant attention 1
- J'y prête autant d'attention que par le passé 0

11 J'ai la bougeotte et n'arrive pas à tenir en place :
- Oui, c'est tout à fait le cas 3
- Un peu 2
- Pas tellement 1
- Pas du tout 0

12. Je me réjouis d'avance à l'idée de faire certaines choses :
- Autant qu'avant 0
- Un peu moins qu'avant 1
- Bien moins qu'avant 2
- Presque jamais 3

13 J'éprouve des sensations soudaines de panique :
- Vraiment très souvent 3
- Assez souvent 2
- Pas très souvent 1
- Jamais 0

14. Je peux prendre plaisir à un bon livre ou à une bonne émission de radio ou de télévision :
- Souvent 0
- Parfois 1
- Rarement 2
- Très rarement 3

45 - Additionnez les points des réponses 1, 3, 5, 7, 9, 11, 13 : Total A =
46 - Additionnez les points des réponses 2, 4, 6, 8, 10, 12, 14 : Total D =

La place du tabac dans votre budget

La dépendance tabagique conduit à accorder une place importante au tabac dans votre budget. Les questions suivantes ont pour objectifs de vous aider à faire le point sur ce que vous dépensez pour le tabac et d'envisager les économies réalisées en arrêtant de fumer.
Les questions 49 à 59 vous permettent d'examiner votre situation actuelle et d'imaginer comment elle pourrait s'améliorer avec l'arrêt du tabac.

47 - Quelle somme d'argent consacrez-vous au tabac chaque semaine ? ____________

48 - Dans quelle tranche se situe la somme totale de vos revenus mensuels nets (y compris les aides sociales) ?

☐ Moins de 1000 € ☐ Entre 1001 et 2000 € ☐ Entre 2001 et 3000 €

☐ entre 3001 et 4000 € ☐ plus de 4000 €

Pensez-y L'Assurance Maladie vous accompagne dans l'arrêt du tabac. Elle prend en charge, sur prescription médicale établie par un médecin ou une sage-femme, les traitements par substituts nicotiniques à hauteur de 50 € par année civile et par bénéficiaire. Pour les femmes enceintes, les jeunes de 20 à 30 ans, les bénéficiaires de la CMU complémentaire et les patients en ALD cancer, ce montant est de 150 €

49 - Rencontrez-vous parfois un travailleur social ? Oui ☐ - Non ☐

50 - Bénéficiez-vous d'une assurance maladie complémentaire ? Oui ☐ - Non ☐

51 - Vivez-vous en couple ? Oui ☐ - Non ☐

52 - Êtes-vous propriétaire de votre logement ? Oui ☐ - Non ☐

53 - Y a-t-il des périodes dans le mois où vous rencontrez de réelles difficultés financières à faire face à vos besoins (alimentation, loyer, EDF…) ? Oui ☐ - Non ☐

54 - Vous est-il arrivé de faire du sport au cours des 12 derniers mois ? Oui ☐ - Non ☐

55 - Êtes-vous allé(e) au spectacle au cours des 12 derniers mois ? Oui ☐ - Non ☐

56 - Êtes-vous parti(e) en vacances au cours des 12 derniers mois ? Oui ☐ - Non ☐

57 - Au cours des 6 derniers mois, avez-vous eu des contacts avec des membres de votre famille autres que vos parents ou vos enfants ? Oui ☐ - Non ☐

En cas de difficultés, y a-t-il des personnes dans votre entourage sur qui vous pouvez compter

58 - pour vous héberger quelques jours ? Oui ☐ - Non ☐

59 - pour vous apporter une aide matérielle ? Oui ☐ - Non ☐

Les questions 49 à 59 font partie du questionnaire EPICES, mis au point dans les centres d'examens de santé afin d'évaluer votre situation sociale.

Les renseignements recueillis dans de ce document seront traités ultérieurement de manière anonyme par l'équipe CDTnet, dans le cadre d'un recueil national. L'équipe CDTnet a pour objectif d'améliorer la prise en charge de toute personne souhaitant arrêter de fumer.
https://cdtnet.spim.jussieu.fr

Dans quelques mois, vous êtes susceptible de recevoir une lettre, un mail ou un appel téléphonique afin de savoir si vous êtes encore fumeur ou non. Ces informations sont importantes car il s'agit pour nous d'apprécier l'aide qui a pu vous être apportée.

Merci d'avance de répondre à cette lettre ou cet appel téléphonique.

Si vous préférez être contacté à ce sujet par mail, merci de nous indiquer votre adresse e-mail :
______________________ @ __________________________

Si vous ne souhaitez pas être recontacté à ce sujet, cochez la case suivante ☐

Cette page sera remplie par le médecin, merci de ne rien inscrire.

Bilan lors de la consultation initiale

Date : |__|__| |__|__| |__|__|
jour mois année

60 - Consommation moyenne quotidienne de tabac : _______

61 - CO expiré : |__|__| ppm

62 - Nombre de cigarettes depuis le lever : |__|__|

63 - Temps écoulé depuis la dernière cigarette : ___________ minutes/heures/jours

64 - **Observations :**

Conclusions :

65 - Une date d'arrêt a-t-elle été fixée avec le fumeur ? Oui ☐ - Non ☐

66 - Démarche de réduction de consommation

☐ Sans traitement dans un premier temps

☐ Avec un traitement prescrit (détail ci-dessous)

67 - Traitement prescrit

- Traitement de substitution nicotinique : Oui ☐ - Non ☐

lequel : ☐ Dispositif transdermique - ou patch : dose par 16 h ou 24 h ___________

☐ Gommes 2 mg 4 mg ☐ Comprimés à sucer 1 mg 1,5 mg 2 mg 2,5 mg 4 mg

☐ Spray buccal ☐ Inhaleurs ☐ Comprimés sublinguaux (microtab 2 mg)

- Autre traitement pharmacologique d'aide au sevrage tabagique :

☐ Bupropion (Zyban®) ☐ Varenicline (Champix®)

- Techniques comportementales : Oui ☐ - Non ☐
- Orientation vers un psychologue ou un psychiatre : Oui ☐ - Non ☐
- Prise en charge diététique : Oui ☐ - Non ☐
- Autre : ______________________________________

Date : ⌊_|_⌋ ⌊_|_⌋ ⌊_|_⌋
jour mois année

Consultation de suivi N° 1

68 - Patient en arrêt : Oui ☐ - Non ☐

Si oui : nombre de jours d'arrêt total : __________

Si non : réduction de consommation de 50 % et plus : Oui ☐ - Non ☐

69 - Consommation moyenne quotidienne de cigarettes : __________

70 - CO expiré : ⌊_|_⌋ ppm

71 - Temps écoulé depuis la dernière cigarette : __________ minutes/heures/jours

72 - Utilisez-vous une cigarette électronique ? Oui ☐ - Non ☐

Pour les utilisateurs de cigarettes électroniques

73 - Quel est le volume de liquide "vapoté" par semaine ? __________

74 - Utilisez-vous parfois des cartouches de nicotine ? Oui ☐ - Non ☐

75 - Si oui : Quel est le dosage des cartouches de nicotine que vous utilisez ?

76 - Utilisez-vous la cigarette électronique
plus souvent que lors de votre précédente consultation ? Oui ☐ - Non ☐

Merci d'inviter votre patient à partager son expérience de « vapotage » sur le site internet https://cdtnet.spim.jussieu.fr afin de contribuer à améliorer les connaissances à ce sujet.

77 - **Observations :**

78 - **Traitement prescrit :**

- Traitement de substitution nicotinique : Oui ☐ - Non ☐

lequel : ☐ Dispositif transdermique - ou patch : dose par 16 h ou 24 h __________

☐ Gommes 2 mg 4 mg ☐ Comprimés à sucer 1 mg 1,5 mg 2 mg 2,5 mg 4 mg

☐ Spray buccal ☐ Inhaleurs ☐ Comprimés sublinguaux (microtab 2 mg)

- Autre traitement pharmacologique d'aide au sevrage tabagique :

☐ Bupropion (Zyban®) ☐ Varenicline (Champix®)

- Techniques comportementales : Oui ☐ - Non ☐
- Orientation vers un psychologue ou un psychiatre : Oui ☐ - Non ☐
- Prise en charge diététique : Oui ☐ - Non ☐
- Autre : ______________________________

Cette page sera remplie par le médecin, merci de ne rien inscrire.

Date : |_|_| |_|_| |_|_|
jour mois année

Consultation de suivi N° 2

79 - Patient en arrêt : Oui ☐ - Non ☐

Si oui : nombre de jours d'arrêt total : __________

Si non : réduction de consommation de 50 % et plus : Oui ☐ - Non ☐

80 - Consommation moyenne quotidienne de cigarettes : __________

81 - CO expiré : |_|_| ppm

82 - Temps écoulé depuis la dernière cigarette : __________ minutes/heures/jours

83 - Utilisez-vous une cigarette électronique ? Oui ☐ - Non ☐

Pour les utilisateurs de cigarettes électroniques

84 - Quel est le volume de liquide "vapoté" par semaine ? __________

85 - Utilisez-vous parfois des cartouches de nicotine ? Oui ☐ - Non ☐

86 - Si oui : Quel est le dosage des cartouches de nicotine que vous utilisez ?

87 - Utilisez-vous la cigarette électronique
plus souvent que lors de votre précédente consultation ? Oui ☐ - Non ☐

Merci d'inviter votre patient à partager son expérience de « vapotage » sur le site internet https://cdtnet.spim.jussieu.fr afin de contribuer à améliorer les connaissances à ce sujet.

88 - **Observations :**

89 - **Traitement prescrit :**

- Traitement de substitution nicotinique : Oui ☐ - Non ☐

  lequel : ☐ Dispositif transdermique - ou patch : dose par 16 h ou 24 h __________

  ☐ Gommes 2 mg 4 mg ☐ Comprimés à sucer 1 mg 1,5 mg 2 mg 2,5 mg 4 mg

  ☐ Spray buccal ☐ Inhaleurs ☐ Comprimés sublinguaux (microtab 2 mg)

- Autre traitement pharmacologique d'aide au sevrage tabagique :

  ☐ Bupropion (Zyban®) ☐ Varenicline (Champix®)

- Techniques comportementales : Oui ☐ - Non ☐
- Orientation vers un psychologue ou un psychiatre : Oui ☐ - Non ☐
- Prise en charge diététique : Oui ☐ - Non ☐
- Autre : ______________________________

Date : |__|__| |__|__| |__|__|
jour mois année

Consultation de suivi N° 3

90 - Patient en arrêt : Oui ☐ - Non ☐

Si oui : nombre de jours d'arrêt total : __________

Si non : réduction de consommation de 50 % et plus : Oui ☐ - Non ☐

91 - Consommation moyenne quotidienne de cigarettes : __________

92 - CO expiré : |__|__| ppm

93 - Temps écoulé depuis la dernière cigarette : __________ minutes/heures/jours

94 - Utilisez-vous une cigarette électronique ? Oui ☐ - Non ☐

Pour les utilisateurs de cigarettes électroniques

95 - Quel est le volume de liquide "vapoté" par semaine ? __________

96 - Utilisez-vous parfois des cartouches de nicotine ? Oui ☐ - Non ☐

97 - Si oui : Quel est le dosage des cartouches de nicotine que vous utilisez ?

98 - Utilisez-vous la cigarette électronique
plus souvent que lors de votre précédente consultation ? Oui ☐ - Non ☐

Merci d'inviter votre patient à partager son expérience de « vapotage » sur le site internet https://cdtnet.spim.jussieu.fr afin de contribuer à améliorer les connaissances à ce sujet.

99 - **Observations :**

100 - **Traitement prescrit :**

- Traitement de substitution nicotinique : Oui ☐ - Non ☐

  lequel : ☐ Dispositif transdermique - ou patch : dose par 16 h ou 24 h __________

  ☐ Gommes 2 mg 4 mg ☐ Comprimés à sucer 1 mg 1,5 mg 2 mg 2,5 mg 4 mg

  ☐ Spray buccal ☐ Inhaleurs ☐ Comprimés sublinguaux (microtab 2 mg)

- Autre traitement pharmacologique d'aide au sevrage tabagique :

  ☐ Bupropion (Zyban®) ☐ Varenicline (Champix®)

- Techniques comportementales : Oui ☐ - Non ☐
- Orientation vers un psychologue ou un psychiatre : Oui ☐ - Non ☐
- Prise en charge diététique : Oui ☐ - Non ☐
- Autre : ______________________________
